# Proteomic Analysis of Human Skin Treated with Larval Schistosome Peptidases Reveals Distinct Invasion Strategies among Species of Blood Flukes

**DOI:** 10.1371/journal.pntd.0001337

**Published:** 2011-09-27

**Authors:** Jessica Ingram, Giselle Knudsen, K. C. Lim, Elizabeth Hansell, Judy Sakanari, James McKerrow

**Affiliations:** 1 Tetrad Graduate Program, University of California San Francisco, San Francisco, California, United States of America; 2 Mass Spectrometry Facility, Department of Pharmaceutical Chemistry, University of California San Francisco, San Francisco, California, United States of America; 3 Sandler Center for Drug Discovery, California Institute for Quantitative Biosciences (QB3), University of California San Francisco, San Francisco, California, United States of America; University of Queensland, Australia

## Abstract

**Background:**

Skin invasion is the initial step in infection of the human host by schistosome blood flukes. Schistosome larvae have the remarkable ability to overcome the physical and biochemical barriers present in skin in the absence of any mechanical trauma. While a serine peptidase with activity against insoluble elastin appears to be essential for this process in one species of schistosomes, *Schistosoma mansoni*, it is unknown whether other schistosome species use the same peptidase to facilitate entry into their hosts.

**Methods:**

Recent genome sequencing projects, together with a number of biochemical studies, identified alternative peptidases that *Schistosoma japonicum* or *Trichobilharzia regenti* could use to facilitate migration through skin. In this study, we used comparative proteomic analysis of human skin treated with purified cercarial elastase, the known invasive peptidase of *S. mansoni*, or *S. mansoni* cathespin B2, a close homolog of the putative invasive peptidase of *S. japonicum*, to identify substrates of either peptidase. Select skin proteins were then confirmed as substrates by *in vitro* digestion assays.

**Conclusions:**

This study demonstrates that an *S. mansoni* ortholog of the candidate invasive peptidase of *S. japonicum* and *T. regenti*, cathepsin B2, is capable of efficiently cleaving many of the same host skin substrates as the invasive serine peptidase of *S. mansoni*, cercarial elastase. At the same time, identification of unique substrates and the broader species specificity of cathepsin B2 suggest that the cercarial elastase gene family amplified as an adaptation of schistosomes to human hosts.

## Author Summary

Schistosome parasites are a major cause of disease in the developing world, but the mechanism by which these parasites first infect their host has been studied at the molecular level only for *S. mansoni*. In this paper, we have mined recent genome annotations of *S. mansoni* and *S. japonicum*, a zoonotic schistosome species, to identify differential expansion of peptidase gene families that may be involved in parasite invasion and subsequent migration through skin. Having identified a serine peptidase gene family in *S. mansoni* and a cysteine peptidase gene family in *S. japonicum*, we then used a comparative proteomic approach to identify potential substrates of representative members of both classes of enzymes from *S. mansoni* in human skin. The results of this study suggest that while these species evolved to use different classes of peptidases in host invasion, both are capable of cleaving components of the epidermis and dermal extracellular matrix, as well as proteins involved in the host immune response against the migrating parasite.

## Introduction

Human skin is a formidable barrier for much of the microbial world. In addition to the mechanical barrier of structural proteins in the epidermis, basement membrane and dermal extracellular matrix, both the epidermis and dermis are bathed in plasma proteins, including early sentinels of the immune system [1]. In order to successfully breach this barrier, an invading pathogen must degrade protein matrices while minimizing the immune response that it elicits. To this end, many invading organisms utilize insect bites or other mechanical trauma to facilitate their entry into skin, but the multi-cellular larvae of the schistosome blood fluke—the causative agent of the disease schistosomiasis—have the remarkable ability to directly penetrate host skin and gain access to dermal blood vessels [2], [3].

The invasive larva(e)—termed cercaria(e)—is 300 µm long, 70 µm wide and comprised of roughly 1000 cells [4]. Upon direct contact with the surface of human skin, cercariae begin to secrete vesicles containing a variety of proteins and an adhesive, mucin-like substance [5]. Proteomic studies identified the majority of proteins secreted by *S. mansoni* cercariae. These include histolytic peptidases [6], [7]. The most abundant peptidase in *S. mansoni* secretions is an S1A serine peptidase, termed cercarial elastase (SmCE) (GenBank: AAC46967.1) that has activity against insoluble elastin and other fibrillar macromolecules of skin [8]. Biochemical and immunolocalization studies have confirmed SmCE activity in cercarial secretions [9], [10]. Moreover, applying an irreversible serine peptidase inhibitor to *ex vivo* skin before exposure to cercariae blocks the majority of larvae from invading, suggesting that this serine peptidase has an essential role in skin penetration [11].

While the serine peptidase, cercarial elastase, plays a key role in *S. mansoni* skin invasion, the zoonotic species *S. japonicum* has no serine peptidases in its larval secretions. *S. japonicum*, however, encodes a number of isoforms of cathepsin B2 (SjCB2) (GenBank: CAA50305.1), a cysteine peptidase, which are secreted by the invading parasite [12]. Moreover, orthologs of SjCB2 have been identified in the cercarial secretions of other, non-human schistosome species, including members of the genus *Trichobilharzia* [13]. This led us to the hypothesis that the primary invasive peptidase differs between schistosome species, with *S. mansoni*, a human-specific schistosome species, utilizing cercarial elastase, and *S. japonicum*, a zoonotic schistosome species, utilizing cathepsin B2. Given that *T. regenti* also appears to utilize cathepsin B2 for skin invasion, these observations suggest that the use of a serine peptidase in invasion is the exception, not the rule, among parasitic schistosomes. The use of cercarial elastase may reflect unique properties required by *S. mansoni* to preferentially infect human hosts.

To confirm that cathepsin B2 is also capable of facilitating skin invasion, we used a proteomic approach to identify potential substrates in host skin, for both *S. mansoni* cercarial elastase and *S. mansoni* cathepsin B2 (SmCB2) (GenBank: CAC85211.2), a close homolog of *S. japonicum* CB2. Although RNAi has been developed as a tool in juvenile and adult schistosome worms, it is currently unavailable for the intramolluscan and cercarial stages of development [14]. We therefore chose to use a proteomic approach to validate the roles of these peptidases in skin invasion.

We found that the vast majority of cleaved proteins resulting from human skin exposure to either purified SmCE or SmCB2 overlap, suggesting that both enzymes are capable of facilitating parasite migration through skin. However, we also identified several potential substrates in skin that appear to be cleaved by only one of the two enzymes. Candidate substrates were further validated by *in vitro* cleavage of purified human skin proteins with either peptidase. Together, these observations suggest that more than one mechanism of skin penetration may have evolved as an adaptation specific to the schistosome-host relationship.

## Methods

### Phylogenetic analysis of schistosome cercarial elastase and cathepsin B2 proteins

To determine the number of cercarial elastase and cathepsin B2 protein isoforms in schistosome species, all full-length protein sequences (*i.e.*, those possessing the full catalytic core of the peptidase) were collected from both GenBank (NCBI) and *S. japonicum* and *S. mansoni* genome annotation websites (Sanger Institute GeneDB). ClustalW (DNA Databank of Japan), was then used to perform multiple sequence alignments and to construct phylogenetic trees. A Blosum protein weight matrix was used to score the alignment, with a gap open penalty of 10, a gap extension penalty of 0.20, and gap distance penalty of 5. Bootstrapping values were calculated using the p-distance method, with a count of 100. The resulting phylogenetic tree was visualized with the program Dendroscope.

### Purification of S. mansoni CB2 and CE

*S. mansoni* cercariae were shed from *Biomphalaria glabrata* using a light induction method as previously described [11]. SmCE activity was purified from lysate as previously described with the following modifications [15]. Cercariae shed from approximately 50 snails were pelleted by centrifugation at 100 rcf for 1 minute and stored at −20°C. One milliliter of pelleted cercariae was resuspended in 5 ml 300 mM sodium acetate, pH 6.5, 0.1% Triton X-100, 0.1% Tween-20, 0.05% NP40, and sonicated for 1 minute at 40% output. Soluble protein was harvested by centrifugation for 15 minutes at 7, 500 rcf, followed by 0.2 µ filtration. Fractions were again measured for SmCE activity against AAPF-pNA (Ala-Ala-Pro-Phe-p-nitroanilide), and active fractions were run on 10% bis-TRIS polyacrylamide gels (Invitrogen, Carlsbad, CA) according to the manufacturer's specifications, and silver stained [16]. For confirmation of protein identification, bands corresponding to the correct molecular weight of SmCE were excised from the gel, and subjected to in-gel trypsin digestion, followed by LC-MS/MS peptide sequencing, described below. Active site titration was performed using the synthetic peptide inhibitor AAPF-CMK (Ala-Ala-Pro-Phe-chloromethylketone).

Recombinant SmCB2 was expressed in *Pichia pastoris* as previously described [17].

Media containing secreted protein underwent 0.2 µ filtration and lyophilization. SmCB2 activity was purified as previously described [17]. Fractions were monitored for SmCB2 activity against 5 µM ZFR-AMC (Z-Phe-Arg-7-amino-4-carbamoylmethylcoumarin) in citrate-phosphate buffer, pH 5.3 supplemented with 4 mM DTT. Enzyme concentration was measured by active site titration using the cysteine peptidase inhibitor CAO74 (N-(L-3-trans-propylcarbamoyloxirane-2- carbonyl)-L-isoleucyl-L-proline).

### Ethics statement

The human skin sample was taken in compliance with protocols approved by the Committee on Human Research at the University of California, San Francisco. Written informed consent was obtained for the operation and use of tissues removed.

### Skin digestion

Excised human skin was stored at −80°C. For digestion experiments, skin was thawed, dissected into eight 150–170 mg sections, and placed in 1.5 ml microfuge tubes. To each of these skin sections 100 µl of digestion solution containing either peptidase or inhibited peptidase at 1.8 µM was added, along with corresponding controls. SmCE reaction buffer consisted of 100 mM TRIS-HCl, pH 8; SmCB2 reaction buffer consisted of 100 mM sodium acetate, pH 5.5, 4 mM DTT. Inhibited SmCE was prepared by incubating 1.8 µM SmCE with 2 µM AAPF-CMK for one hour at room temperature; inhibition was monitored against AAPF-pNA, prior to its addition to skin. Similarly, inhibited SmCB2 digestion solution was prepared by incubating 1.8 µM SmCB2 with 2 µM CAO74 for one hour at room temperature, with full inhibition monitored by activity against ZFR-AMC, prior to its addition to skin. Inhibitor alone digestion solutions were prepared to control for human skin peptidase activity using either 2 µM AAPF-CMK in 100 mM Tris, pH 8.0, or 2 µM CAO74 in 100 mM sodium acetate, pH 5.5, 4 mM DTT. After addition of digestion solution to skin samples, the reaction mix was vortexed briefly, and then incubated for 5 hours at 37°C. Following incubation, reactions were centrifuged for 20 minutes at 16,000 rcf at 4°C, and the resulting supernatant was saved as the soluble fraction. Fifteen microliters were removed for analysis on a bis-TRIS 4-20% acrylamide gel. Gels were silver-stained and stored at 4°C.

### Proteomic/mass spectrometry analysis

Proteomic analysis of skin digestion samples was performed by LC-MS/MS on two independent preparations as follows. Representative preparative gels are shown in Figures S1 and S2, and contain replicate lanes of approximately 20 µg total protein for each of the skin digestion solutions. Each pair of sample lanes was cut into ten protein bands, and diced into 1–2 mm cubes, then subjected to in-gel trypsin digestion, following a previously published protocol [6]. The resulting peptides were extracted and analyzed by on-line liquid chromatography/mass spectrometry using an Eksigent nanoflow pump and a Famos autosampler that were coupled to a quadrupole-orthogonal-acceleration-time-of-flight hybrid mass spectrometer (QStar Pulsar or QStar Elite, Applied Biosystems, Foster City, CA). Peptides were fractionated on a reversed-phase column (C18, 0.75×150 mm) and a 5–50% B gradient was developed in 35 min at a 350 nl/min flow rate. Solvent A was 0.1% formic acid in water, solvent B was 0.1% formic acid in acetonitrile. Data were acquired in information-dependent acquisition mode: 1 sec MS surveys were followed by 3 sec CID experiments on computer-selected multiply charged precursor ions. Peak lists were generated using Analyst 2.0 software (Applied Biosystems) with the Mascot script 1.6b20 (Matrix Science, London, UK).

Database searches were performed using ProteinProspector v. 5.7.1 (http://prospector2.ucsf.edu) [18]. Searches were performed using the SwissProt databank (August 10, 2010, 519,348 entries). For false discovery rate estimation, this database was concatenated with randomized sequences generated from the original database [19]. Search parameters included selecting trypsin as the digestion enzyme, allowing one missed cleavage but no non-specific cleavages. Peptide modifications that were searched included carbamidomethyl (Cys) as the only fixed modification, and up to two variable modifications from among the following: oxidation (Met), acetyl (N-term), oxidized acetyl (N-term), pyroglutamate (Gln), Met-loss (N-term), and Met-loss+acetyl (N-term). Mass accuracy settings were 200 ppm for precursor and 300 ppm for fragment masses. Data reported in Table S3 has a Protein Prospector minimum score cutoff of 22 (protein), 15 (peptide) and maximum expectation values of 0.01 (protein) and 0.05 (peptide), resulting in a 2% false discovery rate.

### Human collagen I and complement C3 cleavage and N-terminal sequencing

Lyophilized type I human skin collagen (Calbiochem) was resuspended in 17.5 mM acetic acid for a final concentration of 1 mg/ml. Human complement C3 (Calbiochem) was purchased as a 1.2 mg/ml stock. For SmCB2 digestion, 180 nM enzyme was added to 50 µl collagen I or 25 µl complement C3 in 50 mM sodium acetate, pH 5.5, 4 mM DTT and incubated at 37°C for 1–22 hours. For SmCE digestion, 180 nM enzyme was added to 50 µl collagen I or 25 µl Complement C3 in 50 mM Tris, pH 8.0 and incubated at 37°C for 1–22 hours. Both enzymes were also pre-incubated with 1 mM CAO74 (SmCB2) or 1 mM AAPF-CMK (SmCE) for one hour at room temperature prior to their addition to collagen. As a control, collagen was incubated in 50 mM sodium acetate, pH 5.5, 4 mM DTT or 50 mM Tris, pH 8.0 for 22 hours at 37°C. To stop the reaction, 15 µl reduced SDS-PAGE loading dye (Invitrogen) was added, and a sample of each reaction was run on a 4–20% Tris-Glycine SDS PAGE gel (Invitrogen). Bands were then electroblotted onto PVDF membrane (Biorad, Foster City, CA) and visualized by Coomassie Blue staining. N-terminal sequence of selected bands was determined using Edman chemistry on an Applied Biosystems Procise liquid-pulse protein sequenator at the Protein and Nucleotide Facility, Stanford University.

## Results

### Identification and phylogeny of cercarial elastase and cathepsin B2 isoforms

**Figure 1:**
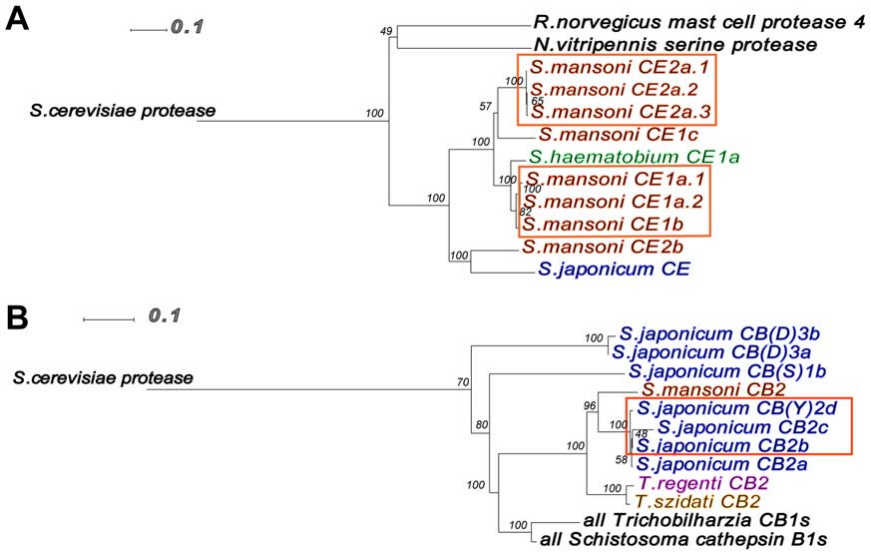
Differential expansion of select peptidase gene families in schistosome species. Phylogenetic analysis of cercarial elastase (A) and cathepsin B2 (B) protein sequences reveals expansion of each gene family in different lineages of schistosomes. Boxes indicate proteins previously determined to be present in cercarial secretions, as determined by LC MS/MS.

To outline the molecular evolution of larval peptidases in schistosomes, all previously reported orthologs were re-examined (Figure 1). In addition to the previously identified full-length cercarial elastase isoforms in *S. mansoni*--SmCE1a (GenBank: AAM43939.1), SmCE1b (GenBank: CAA94312.1), SmCE1c (GenBank: AAC46968.1), SmCE2a (AAM43941.1) and SmCE2b (GenBank: AAM43942.1) and *Schistosoma haematobium* cercarial elastase (GenBank: AAM4394)--sequencing and annotation of the full *S. mansoni* genome revealed three additional full-length genes [15], [20] (Figure 1A). In marked contrast, the *S. japonicum* genome contains only a single cercarial elastase isoform (Sjp_0028090). No cercarial elastase genes have been detected in any *Trichobilharzia* species.

Both *S. mansoni* and *S. japonicum* encode a number of cathepsin B genes (Figure S3). We chose to focus on the cathepsin B2 isotype, since a proteomic analysis of *S. japonicum* cercarial secretions identified a peptide sequence common to this subset [12] (Figure 1B). Notably, while the *S. mansoni* genome encodes only a single cathepsin B2 isoform, *S. japonicum* encodes four CB2 isoforms. In one of these isoforms, SjCB(Y)2d (GenBank: CAX71091.1), the nucleophilic cysteine of the active site is mutated to tyrosine, which may diminish, if not eliminate, its catalytic activity. Three of the four SjCB2 isoforms (SjCB2b (GenBank: CAX71088.1), SjCB2c (GenBank: CAX71090.1) and SjCB(Y)2d correspond to the peptide sequence identified in proteomic analysis of *S. japonicum* cercarial secretions [12]. A full list of schistosome cercarial elastase and cathepsin B isoforms is provided as supplementary material (Tables S1 and S2).

### Comparative proteomic analysis of human skin treated with SmCE and SmCB2

A previous proteomic study generated a list of proteins that were released as soluble peptides from *ex vivo* human skin upon treatment with live *S. mansoni* cercariae, indicating that they are actively degraded during cercarial migration through skin [21]. These included many of the structural components of skin, including extracellular matrix proteins, proteins involved in cell-cell adhesion and multiple serum proteins. To identify specific substrates of CE in skin, and to compare these to potential substrates of cathepsin B2, we treated *ex vivo* skin with either peptidase. Since active, recombinant *S. japonicum* cathepsin B2 is not currently available, and purifying sufficient amounts of native peptidase from *S. japonicum* was not feasible, we used *S. mansoni* cathepsin B2 as model peptidase in our analysis. *S. mansoni* cathepsin B2 has high homology to the *S. japonicum* cathepsin B2 (90% sequence identity and 94% sequence similarity for the mature peptidase, see Figure S4), including the active site and substrate binding pocket, and therefore is likely to display highly similar biochemical properties and substrate specificity [17]. SmCE was purified directly from *S. mansoni* cercariae, and the protein composition of proteolytically active fractions was determined by mass spectrometric analysis as a mixture of SmCE1a, 1b and 2a isoforms, but not SmCE2b. This is consistent with the isoform composition of previous proteomic analysis of *S. mansoni* cercarial secretions [6], [22]. Active SmCB2 was expressed in recombinant form in *P. pastoris* and purified as previously described [17]. To ensure that equimolar amounts of active enzyme were added to skin samples, an active site titration was first performed for both SmCE and SmCB2 with respective covalent inhibitors.

In comparison to control samples treated with inhibited peptidase, multiple skin proteins migrated through an SDS-PAGE gel as smaller fragments, *i.e.* fragments less than the predicted molecular weight of the full-length protein, upon addition of active SmCE or SmCB2. These were thus identified as substrates of the specific enzyme and included multiple extracellular matrix proteins (Table 1). Addition of both SmCE and SmCB2 to skin led to the cleavage of collagen VI, which is found in interstitial tissue, and collagen XII, a collagen located in the basement membrane of the epidermis [23]. Only samples incubated with active SmCB2 showed cleavage of collagens I, III and XVIII. In addition to collagen, several other components of the extracellular matrix were degraded upon treatment with either peptidase, including vitronectin, fibronectin, and galectin. Both vimentin and talin-1, cytoskeletal proteins that are associated with desmosomes, were cleaved upon addition of either peptidase. Two additional extracellular matrix components, tenascin-X and thrombospondin-1, were uniquely cleaved upon addition of SmCB2.

**Table 1:** Substrates of SmCE and SmCB2 identified in *ex vivo* skin.

Category		Substrate of		
Accession Number	Protein Name	SmCE	SmCB2	SmCE and SmCB2
**Extracellular**				
P02741	C-reactive protein		**x**	
P01859	Ig gamma-1 chain C region		**x**	
P01042	Kininogen-1		**x**	
P07996	Thrombospondin-1		**x**	
P17931	Galectin-3	**x**	**x**	**x**
**Extracellular Matrix**				
P07355	Annexin A2		**x**	
P98160	Basement membrane heparan sulfate proteoglycan		**x**	
P16070	CD44 antigen		**x**	
P02452	Collagen alpha-1(I) chain		**x**	
P39060	Collagen alpha-1(XVIII) chain		**x**	
P02751	Fibronectin	**x**	**x**	**x**
P98095	Fibulin-2		**x**	
Q15063	Periostin		**x**	
P24821	Tenascin		**x**	
P22105	Tenascin-X	**x**	**x**	**x**
Q15582	Transforming growth factor-beta-induced protein ig-h3		**x**	
P04004	Vitronectin	**x**	**x**	**x**
P12111	Collagen alpha-1(XII) chain	**x**	**x**	**x**
**Extracellular immune component**				
P01024	Complement C3	**x**	**x**	**x**
P0C0L4	Complement C4-A		**x**	
P00746	Complement factor D		**x**	
P01023	Alpha-2-macroglobulin	**x**	**x**	**x**
**Interstitial tissue**				
P02461	Collagen alpha-1(III) chain		**x**	
P12109	Collagen alpha-1(VI) chain	**x**	**x**	**x**
P12111	Collagen alpha-3(VI) chain	**x**	**x**	**x**
**Keratinocytes**				
P35527	Keratin, type I cytoskeletal 9		**x**	
P31944	Caspase-14		**x**	
**Plasma**				
P01008	Antithrombin-III		**x**	
P00488	Coagulation factor XIII A chain		**x**	
P02671	Fibrinogen alpha chain		**x**	
P00738	Haptoglobin		**x**	
Q14624	Inter-alpha-trypsin inhibitor heavy chain H4		**x**	
P02768	Serum albumin	**x**	**x**	**x**
P07339	Cathepsin D		**x**	

Another subset of extracellular proteins identified as substrates of SmCE and SmCB2 were derived from blood plasma that bathes the dermis. These included components of the coagulation cascade, e. g. fibrinogen, antithrombin-III, as well as proteins involved in the host immune response, e. g. complement C3, complement factor D. Addition of either active SmCE or SmCB2 led to the digestion of gelsolin, an actin assembly protein that exists intracellularly and in plasma. Addition of active SmCB2 also led to the digestion of both kininogen-1 and fibrinogen, both of which are members of the coagulation cascade. Complement C3, an integral component of both the classical and alternative complement activation pathways was cleaved upon addition of either SmCE and SmCB2; complement C4A and complement D proteins, respective members of the classical and alternative complement activation pathways, were cleaved by SmCB2 alone.

In addition to the extracellular proteins identified, many cytosolic proteins were also cleaved by either SmCE or SmCB2. A complete list of peptides identified is provided as a supplementary table (Table S3).

### *In vitro* digestion of human collagen I and complement C3

**Figure 2:**
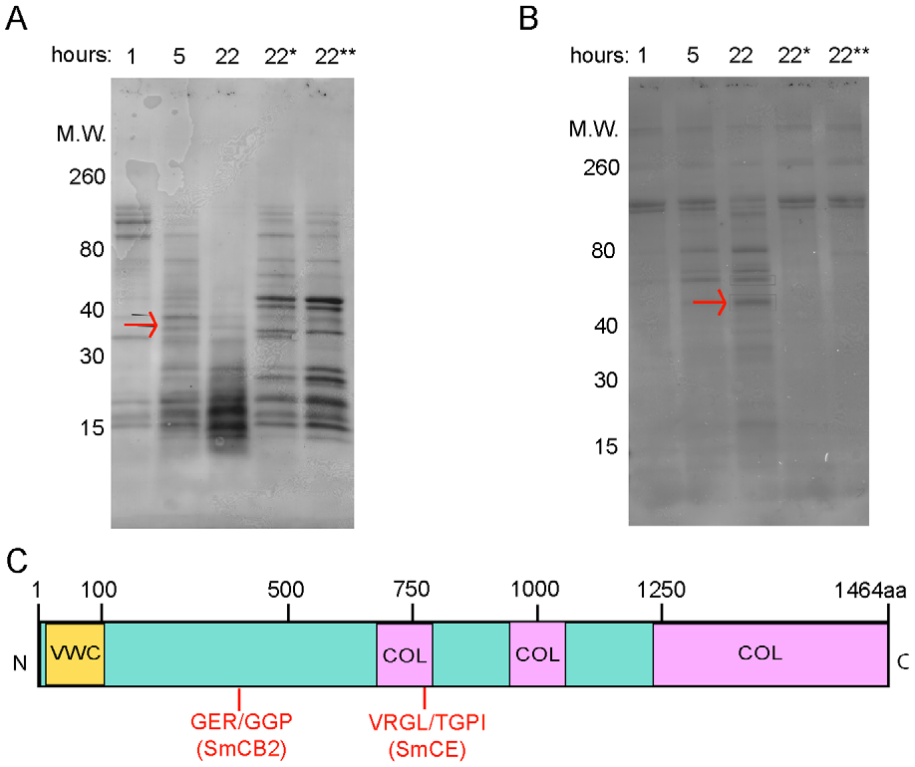
Human collagen I is preferentially cleaved by SmCB2. *In vitro* cleavage of human collagen I confirms *ex vivo* analysis, and reveals differential cleavage by CE and CB2. (A) SmCB2 digestion of collagen I (B) SmCE digestion of collagen I. Digestion reactions were performed for 1–22 hours at 37°C. * indicates pre-incubation with (A) 1 mM CAO74 or (B) 1 mM AAPF-CMK. ** indicates no peptidase control. Arrows indicate bands submitted for Edman degradation. (C) Schematic of human collagen I, indicating cleavage sites as determined by Edman degradation (VWC-Von Willenbrand Factor Type C domain; COL- collagen triple helix repeat).

To corroborate proteomic identification of substrates in skin, candidate substrates were selected for *in vitro* digestion with either SmCE or SmCB2. Type I collagen was of particular interest, given that lower molecular weight peptides of the protein were only found in skin samples treated with SmCB2, suggesting it is cleaved by SmCB2 but not SmCE. To test this with purified protein, type I human collagen was treated with either SmCB2 or SmCE for up to 22 hours at 37°C, and cleavage of the protein was determined by SDS-PAGE analysis (Figure 2). While the majority of collagen I was degraded after 5 hours with SmCB2 (Figure 2A), SmCE treatment resulted in the appearance of discrete lower molecular weight bands only after 22 hours of enzyme treatment (Figure 2B). This confirms that SmCE shows reduced activity against type I collagen relative to SmCB2, even *in vitro*. To confirm that the two peptidases cleaved collagen at unique sites, candidate lower molecular weight bands resulting from peptidase treatment were submitted for N-terminal sequencing, and the resulting amino acid sequence was mapped onto the full protein to determine cleavage sites (Figure 2C). Consistent with previous analysis of SmCE substrate specificity, *in vitro* digestion of collagen I revealed that peptide bond cleavage only occurred following a leucine residue (VRGL/TGPI) [15], [24]. In comparison, SmCB2 cleavage occurred following an arginine residue (GER/GGP), which is consistent with its reported activity, including a level of “promiscuity” in its amino acid preference in the P2 substrate binding pocket, relative to other types of cathepsins [17], [25].

**Figure 3:**
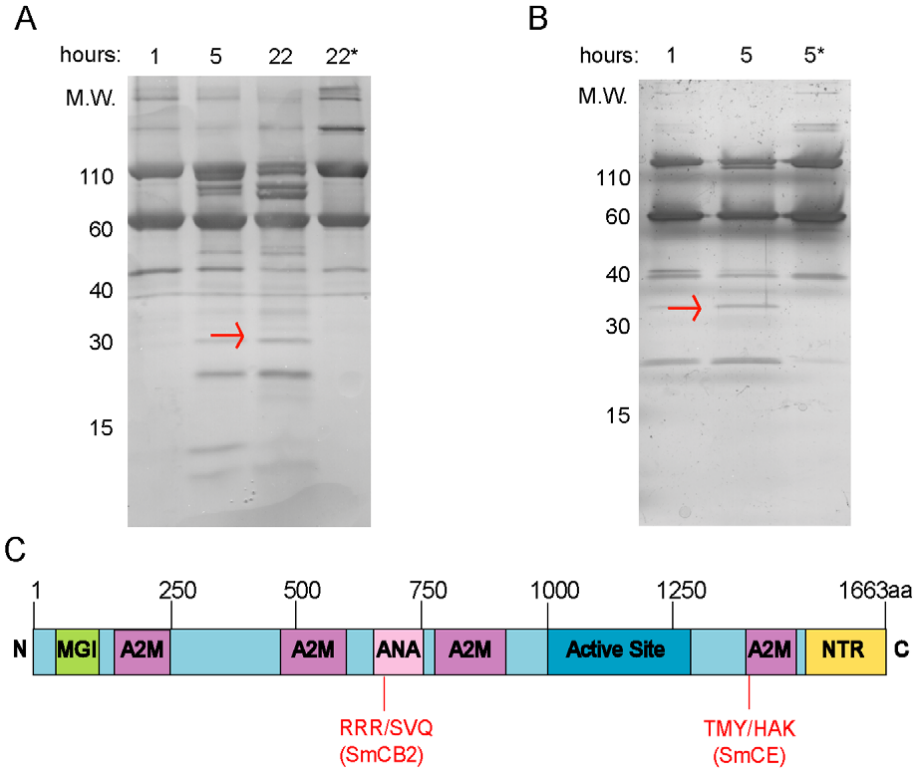
Human complement C3 is cleaved by both SmCE and SmCB2. *In vitro* cleavage of human complement C3 protein confirms *ex vivo* analysis, and reveals differential cleavage by CE and CB2. (A) SmCB2 digestion of complement C3. (B) SmCE digestion of complement C3. Digestion reactions were performed for 1–22 hours (SmCB2) or 1–5 hours (SmCE) at 37°C. * indicates pre-incubation with (A) 1 mM CAO74 or (B) 1 mM AAPF-CMK. ** indicates no peptidase control. Arrows indicate bands submitted for Edman degradation. (C) Schematic of human complement C3, indicating cleavage sites as determined by Edman degradation (MGI-macroglobulin-1; A2M-alpha-2-macroglobulin; ANA-.Anaphylatoxin homologous domain; NTR-netrin-like domain).

Complement C3 was also of particular interest as a potential substrate of both SmCE and SmCB2, given its role in the host immune response against the parasite [26]. Purified complement C3 was treated with SmCB2 or SmCE. Discrete lower molecular weight bands were visible within 1 hour of treatment with either peptidase, in comparison to inhibited peptidase controls (Figure 3A, B). N-terminal sequencing of selected fragments again revealed that both SmCE and SmCB2 digested the protein in a manner consistent with their known specificities, with an arginine in the P1 position (RR/SVQ) for SmCB2 and and a tyrosine in the P1 position (TMY/HAK) for SmCE (Figure 3C).

## Discussion

In *S*. *mansoni*, the most abundant peptidase in cercarial secretions is a serine peptidase, termed cercarial elastase (SmCE) for its ability to degrade insoluble elastin [8], [22]. In addition to proteomic analysis, biochemical and immunolocalization studies have detected SmCE activity in cercarial secretions and confirmed that the enzyme is able to cleave such substrates as type IV collagen (basement membrane collagen), fibronectin, laminin and immunoglobulin *in vitro* [9], [10], [27]. Here, we have shown that SmCE cleaves additional substrates in skin, including several types of collagen, other extracellular matrix proteins, and components of the complement cascade.

Recent sequencing and annotation of the *S. mansoni* genome suggests a unique role for cercarial elastase. An expanded gene family was identified with ten individual genes that encode multiple isoforms of the peptidase. Even without a complete genome, multiple orthologs of SmCE have been also been found in *S. haematobium*, a related human-specific species of schistosome common throughout North Africa and the Middle East [15]. This is not the case for the zoonotic *S. japonicum*, a schistosome species that infects humans and other mammals throughout southeast Asia. The *S. japonicum* genome contains only a single gene encoding cercarial elastase. This gene corresponds to the cercarial elastase “2b” isoform in *S. mansoni*, for which minimal transcript is made relative to other CE isoforms (Ingram and McKerrow, *unpublished*). While one report suggested that CE was detected by immunofluorescence in *S. japonicum* secretions, no cercarial elastase protein was detected in a high resolution mass spectrometric proteomic analysis of *S. japonicum* acetabular secretions, and no cercarial elastase-like activity was identified by direct biochemical assays [12], [20]. *Trichobilharzia regenti*, an avian schistosome that is capable of invading human skin, but not establishing a successful infection in humans, encodes a cysteine peptidase, cathepsin B2 (TrCB2 (GenBank: ABS57370.1)), which has elastinolytic properties and localizes to the acetabular glands of the parasite [13]. *S. japonicum* also encodes a cathepsin B2 ortholog, and transcript is expressed in the developing larval stage of the parasite. Moreover, proteomic analysis has identified cathepsin B2 as being present in *S. japonicum* cercarial secretions [20]. Notably, *S. japonicum* has 40- fold higher cathepsin B activity in its acetabular secretions, relative to *S. mansoni* secretions [12]. It is therefore likely that in *S. japonicum* cercariae, cathepsin B2, not cercarial elastase, is the predominant invasive enzyme.

The differential use of these two classes of peptidases raises the question of how their respective pH optima are achieved in schistosome secretions. SmCB2 is maximally active under acidic, reducing conditions [28]. Since the influence of *S. japonicum* cercarial secretions on the local environment of skin is unknown, SmCB2 incubations were performed under acidic conditions to ensure optimal peptidase activity. SmCE activity is optimal in a slightly alkaline environment, and *S. mansoni* secretions are also alkaline; therefore all SmCE incubations were performed at pH 8 [29]. Certainly, for *S. mansoni*, the evolutionary selection is most likely coordination of the pH of the acetabular gland secretions and the pH optimum of the peptidase. The pH optimum of the cercarial elastase is 8, and the pH of the secretions is also alkaline [30]. As *S. mansoni* cercariae migrate through skin, a microenvironment is created by the secreted material, which allows for optimal activity of the peptidase. The situation is less clear for *S. japonicum* and the *Trichobilharzia* cercariae. While some activity of the cathepsin B2 is likely to continue at neutral, or even alkaline pH, the pH optimum is slightly acidic [17]. The situation is reminiscent of the secretion of cathepsin B by macrophages into tissue compartments of vertebrates. Secreted human cathepsin B is known to degrade extracellular matrix proteins in human tissue, where it has been reported to facilitate tumor invasion and metastasis [31]. The pH optimum of mammalian cathepsin B is also slightly acidic [32]. It is not known if the microenvironment around migrating macrophages is acidic or when that enzyme is released; however, it appears that there is sufficient cathepsin B activity to cause tissue degradation.

Given the unavailability of active, recombinant SjCB2 or sufficient amounts of *S. japonicum* cercariae from which to purify the native enzyme, we chose to perform our proteomic study with SmCB2, which displays high sequence homology (90% amino acid sequence identity for the mature peptidase) to its *S. japonicum* ortholog. We therefore hypothesized that it is likely to display similar biochemical characteristics, including similar substrate specificity. While we cannot say conclusively that SjCB2 is the protease facilitating *S. japonicum* cercarial invasion, we believe that our study, along with previous work from other groups, supports the proposed role for cathepsin B2 in host skin protein degradation [12], [13].

This conclusion, that *S. japonicum* uses a cathepsin B2 peptidase for skin invasion, while *S. mansoni* uses a serine peptidase (SmCE), has implications for the evolution of the human host-parasite relationship in schistosomiasis. A plausible model is that the cathepsin B2 family first emerged as the functional cercarial peptidase during trematode evolution. In contrast, the “humanized” parasites such as *S. mansoni* appear to have switched to a serine peptidase for cercarial invasion. This model is supported by the notable expansion of the serine peptidase gene family from the single 2b gene found in *S. japonicum* to the multiple isoforms expressed in *S. mansoni* [20], [33]. While the genome of the other “humanized” parasite, *S. haematobium*, has not been completed, it is already clear from EST analysis that more abundant serine peptidase isoforms are present in that genome [15].

What is the advantage of a larval serine peptidase for the “humanized” schistosomes? It is interesting to note that by BLAST analysis, some of the proteins with highest homology to cercarial elastase are mammalian mast cell peptidases, which are present in skin [12]. It is therefore possible that cercarial elastase evolved by convergence to resemble a human peptidase, in order to evade detection by the host immune system. Previous work shows that *S. mansoni* cercariae migrate through skin at a much slower rate than their *S. japonicum* counterparts [34]. Despite this, an inflammatory response to *S. japonicum* cercariae occurs more frequently than to *S. mansoni* cercariae [34], [35]. Cathepsin B2 is a likely target of the inflammatory response, given that many cysteine peptidases are allergenic [36]. Perhaps the rapid transit of non-humanized cercariae through skin precludes the need for an invasive enzyme that mimics a host peptidase. Other aspects of immune evasion, such as the elimination of complement factors and immunoglobulin, may be common to both species. C3 and C4 components bind to the tegument of schistosomes, but are degraded by both SmCE and SmCB2 [26], [37].

The results reported here show that S. *mansoni* cathepsin B2 (a model for *S. japonicum* cathepsin B2) and *S. mansoni* cercarial elastase are both capable of degrading proteins in skin that act as a barrier to cercarial invasion. Many skin proteins are substrates for both enzymes, but cathepsin B2 appears to cleave a broader range of substrates, and therefore may be a more effective invasive enzyme than cercarial elastase.

## Supporting Information

Figure S1 **Preparative and analytical SmCE SDS-PAGE gels.** (A) Preparative SDS-PAGE gel with duplicate lanes loaded with skin lysate, treated with (1) 180 nM SmCE, (2) 1.8 µM SmCE, (3) 2 µM AAPF-CMK followed by 2 µM SmCE, or (4) no enzyme. (B) The same samples loaded at the analytical scale, at 1/10 concentration compared to (A).
(TIF)

Figure S2 **Preparative and analytical SmCB2 SDS-PAGE gels.** (A) Preparative SDS-PAGE gel with duplicate lanes loaded with skin lysate, treated with (1) 180 nM SmCB2, (2) 1.8 µM SmCB2, (3) 1.8 µM CA074+1.8 µM SmCB2, or (4) 1.8 uM CA074. (B) The same samples loaded at the analytical scale, at 1/10 concentration compared to (A).
(TIF)

Figure S3 **Expanded phylogenetic analysis of all known schistosome cathepsin B proteins.**
(EPS)

Figure S4 **SmCB2 and SjCB2 protein alignment.** ClustalW alignment of SmCB2 (GenBank: CAZ31207.1) and SjCB2 (GenBank: AAO59414.2) protein sequences shows high sequence identity.
(EPS)

Table S1 **Complete list of schistosome cathepsin B sequences.** All available schistosome cathepsin B isoforms from *S. mansoni* and *S. japonicum* were compiled from GenBank and their respective annotation websites. For each, the name used for this study, previous identifiers, and the full amino acid sequence are listed. Active site residues are highlighted in red. *S. mansoni* sequences are highlighted in pink, and *S. japonicum* sequences are highlighted in blue.
(XLS)

Table S2 **Complete list of schistosome cercarial elastase sequences.** All available full-length sequences (*i. e.* those possessing the full catalytic core) cercarial elastase sequence from *S. mansoni*, *S. haematobium* and *S. japonicum* were compiled from GenBank and their respective annotation websites. For each, the name used for this study, previous identifiers, and the full amino acid sequence are listed. Active site residues are highlighted in red. *S. mansoni* sequences are highlighted in pink, *S. haematobium* seqeuences are highlighted in purple and *S. japonicum* sequences are highlighted in blue.
(XLS)

Table S3 **Complete list of proteins identified in proteomic analysis of skin treated with schistosome peptidases.** Two replicate experiments are shown. For each protein, the accession number, number of unique peptides, percent coverage, and respective scores are listed (detail of how scores were calculated is described in Methods), along with species name and protein name. Samples were compared based on the number of unique peptides for each experimental condition.
(XLS)
