# Supplementary figures and images for: Proteomic Analysis of Human Skin Treated with Larval Schistosome Peptidases Reveals Distinct Invasion Strategies among Species of Blood Flukes
Source: PLoS Negl Trop Dis. 2011 Sep 27;5(9):e1337. doi: 10.1371/journal.pntd.0001337 (PMC3181243; doi:10.1371/journal.pntd.0001337)

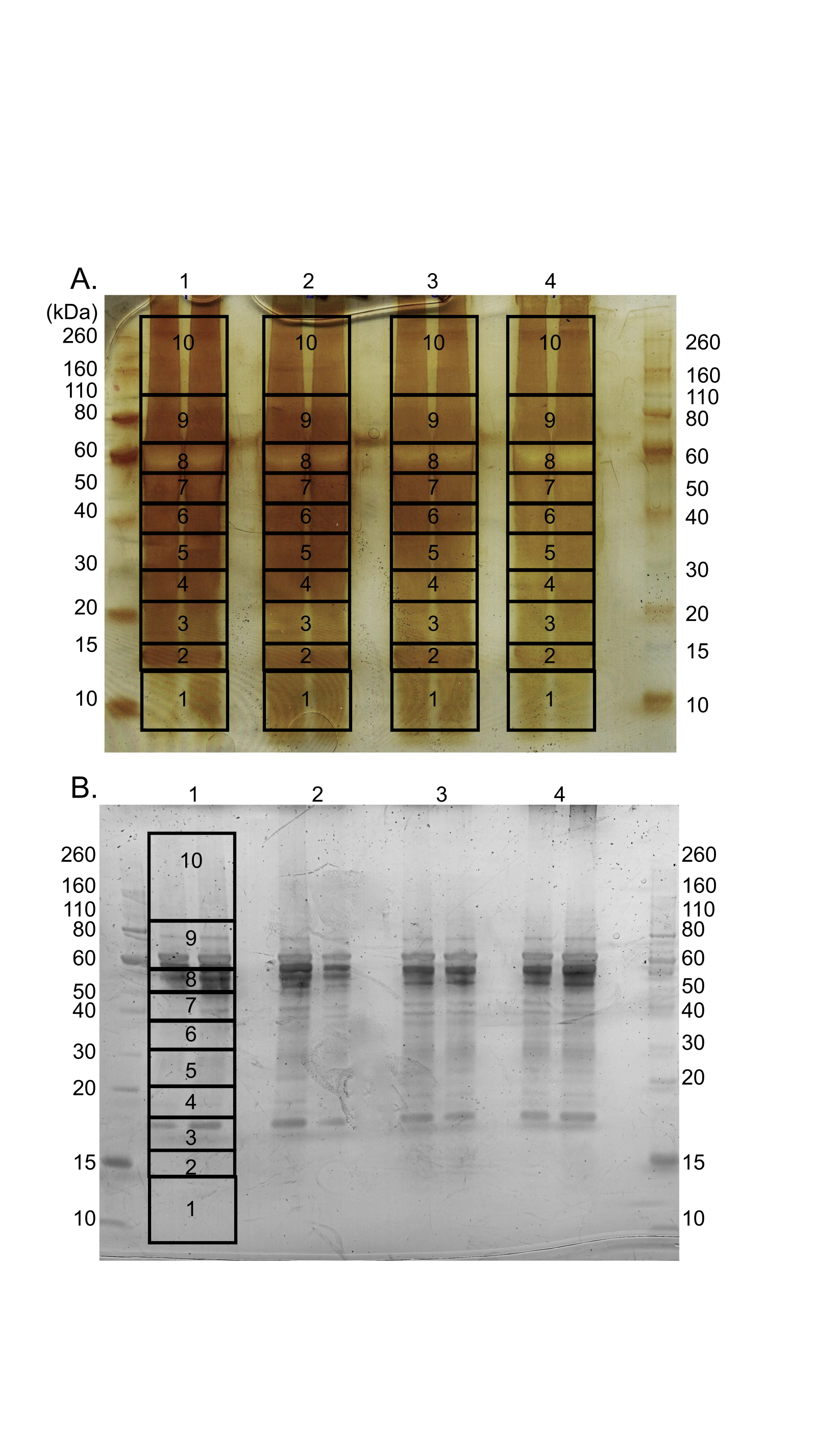

Supplement: Figure S1 — Preparative and analytical SmCE SDS-PAGE gels. (A) Preparative SDS-PAGE gel with duplicate lanes loaded with skin lysate, treated with (1) 180 nM SmCE, (2) 1.8 µM SmCE, (3) 2 µM AAPF-CMK followed by 2 µM SmCE, or (4) no enzyme. (B) The same samples loaded at the analytical scale, at 1/10 concentration compared to (A). (TIF) [file pntd.0001337.s001.tif]

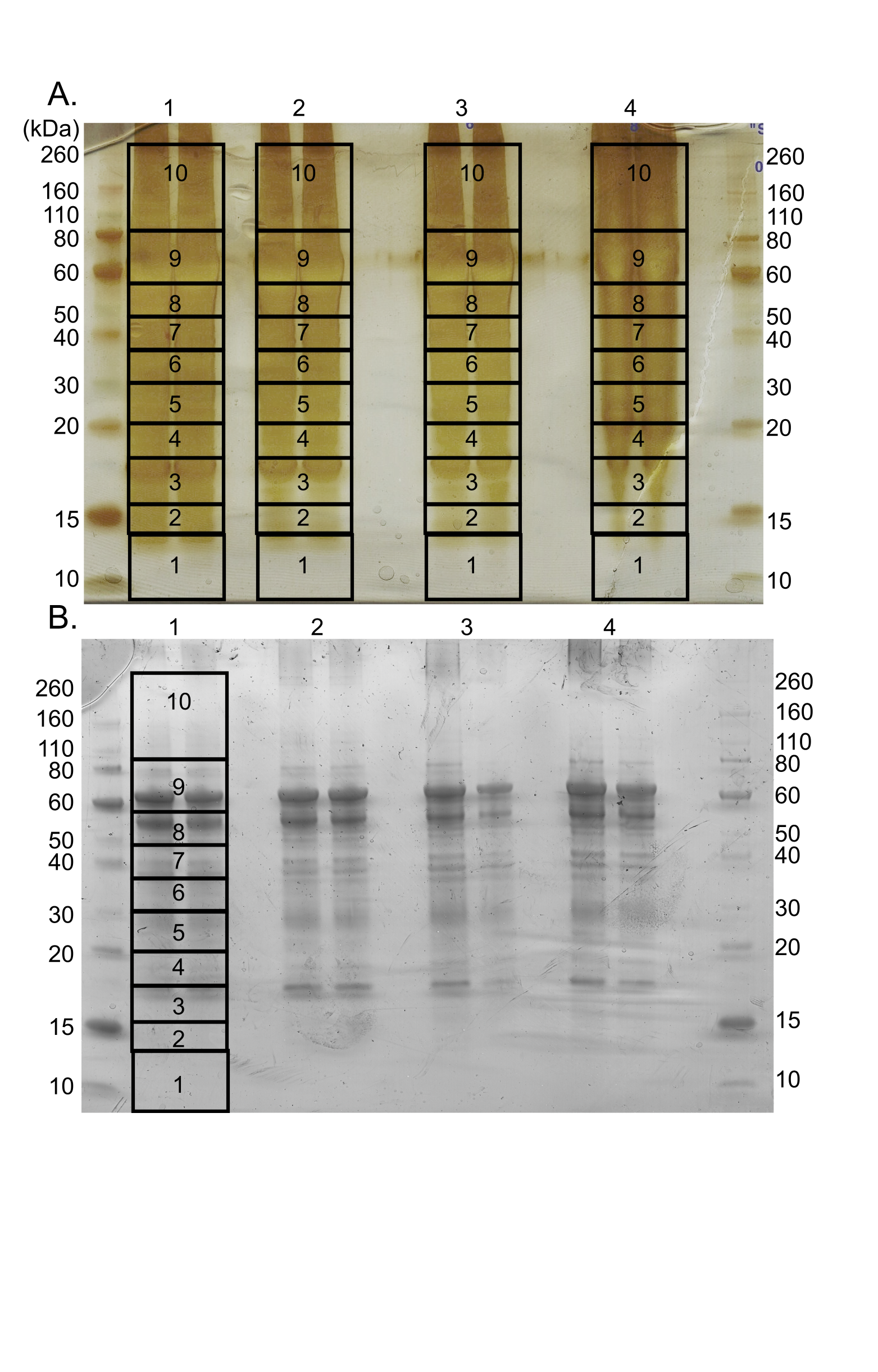

Supplement: Figure S2 — Preparative and analytical SmCB2 SDS-PAGE gels. (A) Preparative SDS-PAGE gel with duplicate lanes loaded with skin lysate, treated with (1) 180 nM SmCB2, (2) 1.8 µM SmCB2, (3) 1.8 µM CA074+1.8 µM SmCB2, or (4) 1.8 uM CA074. (B) The same samples loaded at the analytical scale, at 1/10 concentration compared to (A). (TIF) [file pntd.0001337.s002.tif]
